# Relationships between traditional larval indices and meteorological factors with the adult density of *Aedes albopictus* captured by BG-mosquito trap

**DOI:** 10.1371/journal.pone.0234555

**Published:** 2020-06-11

**Authors:** Jin-Na Wang, Juan Hou, Jian-Yue Zhong, Guo-Ping Cao, Zhang-You Yu, Yu-Yan Wu, Tian-Qi Li, Qin-Mei Liu, Zhen-Yu Gong

**Affiliations:** 1 Zhejiang Provincial Center for Disease Control and Prevention, Hangzhou, China; 2 Quzhou Center for Disease Control and Prevention, Quzhou, China; Faculty of Science, Ain Shams University (ASU), EGYPT

## Abstract

**Objectives:**

Larval indices have been used for *Ae*. *albopictus* surveillance for many years, while there is limited use in assessing dengue transmission risk and adult mosquito emergence. This study is aimed to explore the relationships between larval indices and the *Ae*. *albopictus* density captured by BG-mosquito trap (BG-trap) method, with considering the meteorological factors.

**Methods:**

Data on larval density, adult mosquito density and meteorology factors were collected in an entomological survey carried out in Quzhou City, Zhejiang Province of China in 2018. The Spearman’s rank correlation and Pearson correlation were used for the analysis on the correlation of density indices. Generalized additive models were established to analyze the influencing factors of mosquito density.

**Results:**

Breteau index (BI), House index (HI) and Container index (CI) were highly correlated with each other (r>0.7, p<0.05). The *Ae*. *albopictus* density was significantly correlated with CI (rs = 0.260, p<0.05), CI pre one week (rs = 0.259, p<0.05), and CI pre three weeks (rs = 0.329, p<0.05). BI was correlated with female *Ae*. *albopictus* density pre 4 weeks (r = -0.299, p<0.05). Female *Ae*. *albopictus* density was correlated with CI pre 3 weeks (rs = 0.303, p<0.05). The influencing factors of BI were average wind speed pre 1 week, average temperature and female *Ae*. *albopictus* density pre 4 weeks. The influencing factors of CI were average humidity pre 3 weeks and average temperature. The influencing factors of HI were average temperature and precipitation pre 4 weeks. The influencing factor of *Ae*. *albopictus* density and female *Ae*. *albopictus* density was temperature.

**Conclusions:**

The adult *Ae*. *albopictus* density had low correlation with certain larval indices. Some of the meteorology factors played significant roles in the density of adult *Ae*. *albopictus* and larva with or without a time lag.

## Introduction

*Aedes albopictus* (Skuse), the Asian tiger mosquito, is one of the most invasive insect species in the world with substantial biting activity and high disease vector potential [1]. *Aedes albopictus* is originally from East Asia and the islands of the Pacific and Indian Ocean, and now can be found in all continents except for Antarctica [2]. Worldwide, in over 100 countries of the tropics and subtropics, dengue fever is mainly transmitted by *Ae*. *aegypti* and *Ae*. *albopictus* [3–4]. It is estimated that there are 390 million dengue infections each year globally, and among which, 96 million can produce clinical disease, leading to heavy disease burden [5]. *Ae*. *albopictus* plays a crucial role in the transmission and reservation of dengue virus, not only because dengue virus can circulate in a horizontal transmission (human-mosquito-human), but also it can be transmitted vertically from adult mosquito to offspring [6], which is considered to be a coping mechanism to maintain the virus level under adverse conditions. In the absence of vaccinations and effective drugs, the prevention and control of dengue fever are still focused on the elimination of the mosquito populations [7–8]. From this perspective, developing appropriate strategies to monitor and control the *Ae*. *albopictus* populations should be a priority.

The BG-mosquito trap (BG-trap), which mimics convection currents created by human bodies and releases attractants through a large surface area, can catch significantly more *Ae*. *albopictus* than the Center for Disease Control and Prevention (CDC) light trap and Fay-Prince traps in laboratory settings and field trials [9–10]. Besides, BG-trap is not subjected to the ethical problems of the human landing catch methods, and has a higher efficiency than the human-baited double net trap [11–12]. Since BG-trap is an effective method in monitoring adult *Ae*. *albopictus*, until now, there are no thresholds of the BG-trap that can be served either as reference to determine the timing and intensity of control activities or as a surrogate of dengue transmission risk. The larval indices such as Breteau index (BI), House index (HI) and Container index (CI), are considered more qualitative and often used in *Ae*. *albopictus* density assessment in dengue control [13]. Especially the BI, establishing a relationship between positive containers and houses, is considered the best indices for predicting dengue fever risk [7]. However, incongruences have been found between those larval indices, with the facts that they are limited used in assessing dengue transmission risk and have a poor proxy for measuring adult mosquito emergence [14]. Studies have found that the larval indices are not in accordance with one another or with adult mosquito infestation [13–14], and the strength of correlations between larva and adult populations may depend on season, year, or geographic location [7]. Nevertheless, the relationships of larval indices with adult *Ae*. *albopictus* collected by the BG-trap are still uncertain, and more research is needed to confirm.

Based on previous reports, some meteorological features such as temperature, precipitation and humidity significantly influence the development, as well as survival, density and oviposition rate of mosquito [15–17], which have been found to be associated with dengue fever [18–19]. Among the meteorological features, temperature is considered the main fixed factor driving mosquito development rate, to the exclusion of other factors of known importance such as diet and density [20–21]. Dengue transmission cannot be explained by mosquito density alone, while infection rates and meteorological features should also be considered [15, 22].

In 2018, an entomological survey was carried out in the Quzhou City, Zhejiang Province of China, which provided us an opportunity to study the relationships between traditional larval indices and the adult mosquito density monitored by the BG-trap, with considering the meteorological features.

## Materials and methods

### Study sites and field work

This study was conducted in Quzhou City, Zhejiang Province, located in Southeast China. Considering the aspects of environment, coordination and operability, the Chongwen village (28°53’46.68”N, 118°54’44.02”E) and Songyuan village (28°55’0.37”N, 118°54’15.57”E) exhibited good representatives of the general rural areas in Zhejiang Province and were selected as the study sites. Besides, no major epidemics of dengue fever have occurred in this area during the study period, which could minimize the mosquito density fluctuation for dengue controlling. The study was conducted from April 26 to November 23, 2018 and lasted for 31 weeks. The larval density was monitored in about 50 households every week in Chongwen and Songyuan village, respectively. Trained field workers inspected and recorded household water containers and collected any pupae or larvae present for entomological examination. The water containers included any container with water in or around the households, such as flower pots, water storage containers, idle containers, waste tyres, garbage, rockery pool, open channel, bamboo or tree holes, stone holes, standing water in basement and parking lot, etc. A container was considered positive if it contained at least one larva or pupa.

The BG-trap (model: BG-Mosquitaire CO_2_, Biogents AG, Germany) baited with a steel cylinder filled with CO_2_ emitted at a rate of 500g/24h. The trap was placed on the ground, the BG-Lure (Biogents AG, Germany) was placed in the pocket designated for the lure inside the trap, and the steel cylinder was set next to the trap. Each village placed three BG-traps at the peak time of *Ae*. *albopictus* with more than 200 meters away from each other, and lasted half an hour. All the captured mosquitoes were collected, and the species were identified morphologically.

The larval density and the adult mosquito density were defined as follows [7]. HI: the percentage of houses with containers positive for *Ae*. *albopictus* larvae. CI: the percentage of water-holding containers infested with *Ae*. *albopictus* larvae. BI: the number of positive containers per 100 houses inspected. The *Ae*. *albopictus* density: the number of *Ae*. *albopictus* including male and female trapped per trap in one hour. The female *Ae*. *albopictus* density: the number of female *Ae*. *albopictus* trapped per trap in one hour. Our filed work has been approved by the ethics committee of Zhejiang Provincial Center for Disease Control and Prevention (CDC). The ethics committee approved the procedure for verbal consent because Zhejiang CDC has the authority of the Zhejiang provincial government to collect the related information, which is part of the disease surveillance work in Zhejiang CDC. All the households were notified that they have the right to refuse or terminate the study at any point. Because we obtained verbal consent, documentation of consent was not required. However, the information collected from each household was kept confidential in Zhejiang CDC.

### Meteorological data

The daily meteorological data were collected from National Meteorological Science Data Center, which included precipitation (0.1mm), average air pressure (0.1hpa), average humidity (1%), sunshine hours (0.1h), average temperature(0.1°C), and average wind speed (0.1m/s), etc.

### Statistical analyses

The statistical analyses were conducted with Statistical Program for Social Sciences 21.0 software (SPSS, Inc., Chicago, IL, USA) and R 3.6.2 software (The R Foundation for Statistical Computing Platform). A value of *P*<0.05 was considered as statistically significant. All the parameters were tested for normality. The Spearman’s rank correlation and Pearson correlation with or without time-lag were used to analyze the correlation of the larval density, the adult mosquito density and the meteorological factors according to the data distribution. Generalized additive model (GAM) was used to analyze the influencing factors of the mosquito density.

## Results

### The general description of the water containers

A total of 3109 households were investigated in the study, of which 1491 households had positive water containers, with a positive rate of 47.96%. 8911 water containers were inspected, 3350 was positive and the positive rate was 37.59%. In the positive containers, the highest percentage was seen in *Ae*. *albopictus* (2682, 80.06%), and followed by *Culex pipiens pallens* (631, 18.84%) and *Armigeres obturbans* (37, 1.10%). The BI ranged from 20.00 to 223.53 and the mean value of the two villages was 86.25. The mean value of CI was 30.46%, ranging from 5.52% to 66.13%. The mean value of HI was 42.79%, ranging from 18.00% to 76.00%.

Among all the water containers, the highest proportion was idle containers (6991, 78.45%), and followed by water storage containers (1510, 16.95%). Among different water containers, the highest positive rate was from tire water (48.34%), and followed by garbage water (47.62%) (Table 1).

**Table 1:** Water containers inspected in Chongwen and Songyuan village.

		Chongwen	Songyuan	Total
Flower pots	N	120	41	161
	n	30	21	51
	Positive rate n/N (%)	25.00	51.22	31.68
Water storage containers	N	926	584	1510
	n	275	280	555
	Positive rate n/N (%)	29.70	47.95	36.75
Idle containers	N	3527	3464	6991
	n	1256	1374	2630
	Positive rate n/N (%)	35.61	39.67	37.62
Waste tyres	N	131	80	211
	n	78	24	102
	Positive rate n/N (%)	59.54	30.00	48.34
Garbage	N	12	9	21
	n	3	7	10
	Positive rate n/N (%)	25.00	77.78	47.62
Other containers	N	12	5	17
	n	0	2	2
	Positive rate n/N (%)	0.00	40.00	11.76
Total water containers	N	4728	4183	8911
	n	1642	1708	3350
	Positive rate n/N (%)	34.73	40.83	37.59

N: Number of water containers inspected.

n: Number of positive water containers.

### Adult mosquitoes captured by BG-traps

A total of 680 adult mosquitoes were captured by BG-traps, including 586 (86.18%) *Ae*. *albopictus*, 86 (12.65%) *Ar*. *obturbans*, and 8 (1.18%) *Culex pipiens pallens*. Of all the *Ae*. *albopictus*, 483 (82.42%) were females, and 103 (17.58%) were males. In Chongwen and Songyuan village, 438 and 242 adult mosquitoes were captured, accounting for 64.41% and 35.59%, respectively.

### Correlation between larval density and adult mosquito density

The correlation between the larval density and the adult mosquito density of *Ae*. *albopictus* was analyzed. Considering the possible effect of time lag, 1~4 weeks were selected as the lag effect period. The *Ae*. *albopictus* density was correlated with CI (rs = 0.260, *p* = 0.041), CI pre 1 week (rs = 0.259, *p* = 0.046), and CI pre 3 weeks (rs = 0.329, *p* = 0.013). BI was correlated with female *Ae*. *albopictus* density pre 4 weeks (r = -0.299, *p* = 0.028). Female *Ae*. *albopictus* density was correlated with CI pre 3 weeks (rs = 0.303, *p* = 0.023). The three indices of larval density were highly correlated with each other (the r for BI and CI was 0.741, for BI and HI was 0.916, for CI and HI was 0.753, respectively, *P*<0.05), and were also correlated with a lag effect of 1~4weeks, with correlation coefficients decreased gradually over time.

### Correlations between mosquito density and meteorological factors

The correlation analysis was carried out to explore the relationships between meteorological factors and mosquito density, and 1~4 weeks was selected as the lag effect period. The results showed that the meteorological factors such as precipitation, average air pressure, average humidity, sunshine hours, average temperature, and average wind speed were correlated with different indices of the mosquito density, with or without a lag effect. The significant parameters of the correlation were shown in Table 2.

**Table 2:** The correlations between the mosquito density and the meteorological factors.

	BI		CI		HI		*Ae*. *albopictus* (male and female)		*Ae*. *albopictus* (female)	
	r/rs	P	r/rs	P	r/rs	P	rs	P	rs	P
Average air pressure	-0.424*	0.001	-0.650*	<0.001	-0.448*	<0.001	-0.535	<0.001	-0.516	<0.001
Average temperature	0.354*	0.005	0.622*	<0.001	0.418*	0.001	0.561	<0.001	0.531	<0.001
Precipitation pre 1week	0.329	0.009	—	—	0.277	0.029	—	—	—	—
Average air pressure pre 1 week	-0.392*	0.002	-0.577*	<0.001	-0.445*	<0.001	-0.554	<0.001	-0.534	<0.001
Sunshine hours pre 1 week	—	—	—	—	—	—	0.441	<0.001	0.427	<0.001
Average temperature pre 1 week	0.270*	0.034	0.548*	<0.001	0.352*	0.005	0.581	<0.001	0.563	<0.001
Average wind speed pre1 week	-0.264*	0.038	—	—	—	—	0.278	0.029	0.275	0.030
Precipitation pre 2 weeks	0.445	<0.001	—	—	0.417	0.001	—	—	—	—
Average air pressure pre 2 weeks	-0.336*	0.008	-0.529*	<0.001	-0.416*	0.001	-0.552	<0.001	-0.540	<0.001
Sunshine hours pre 2 weeks	—	—	—	—	—	—	0.351	0.005	0.389	0.002
Average temperature pre 2 weeks	—	—	0.521*	<0.001	0.272*	0.032	0.619	<0.001	0.629	<0.001
Average wind speed pre 2 weeks	—	—	—	—	—	—	—	—	0.278	0.029
Precipitation pre 3 weeks	0.484	<0.001	0.435	<0.001	0.463	<0.001	—	—	—	—
Average air pressure pre 3 weeks	-0.273*	0.032	-0.556*	<0.001	-0.383*	0.002	-0.567	<0.001	-0.551	<0.001
Average humidity pre 3 weeks	—	—	0.369*	0.003	—	—	—	—	—	—
Sunshine hours pre 3 weeks	—	—	—	—	—	—	0.333	0.008	0.350	0.005
Average temperature pre 3 weeks	—	—	0.442*	<0.001	—	—	0.595	<0.001	0.589	<0.001
Average wind speed pre 3 weeks	—	—	—	—	—	—	—	—	0.256	0.045
Precipitation pre 4 weeks	0.583	<0.001	0.400	0.001	0.561	<0.001	—	—	—	—
Average air pressure pre 4 weeks	—	—	-0.490*	<0.001	—	—	-0.606	<0.001	-0.609	<0.001
Average humidity pre 4 weeks	—	—	0.252*	0.048	—	—	—	—	—	—
Average temperature pre 4 weeks	—	—	0.363*	0.004	—	—	0.607	<0.001	0.621	<0.001

All the parameters listed in Table 2 were significant (*P*<0.05).

*Stands for r (the correlation coefficient of the Pearson correlation), and the rest values were rs (the correlation coefficient of the Spearman’s rank correlation).

### The results of the GAM models

**Fig 1:**
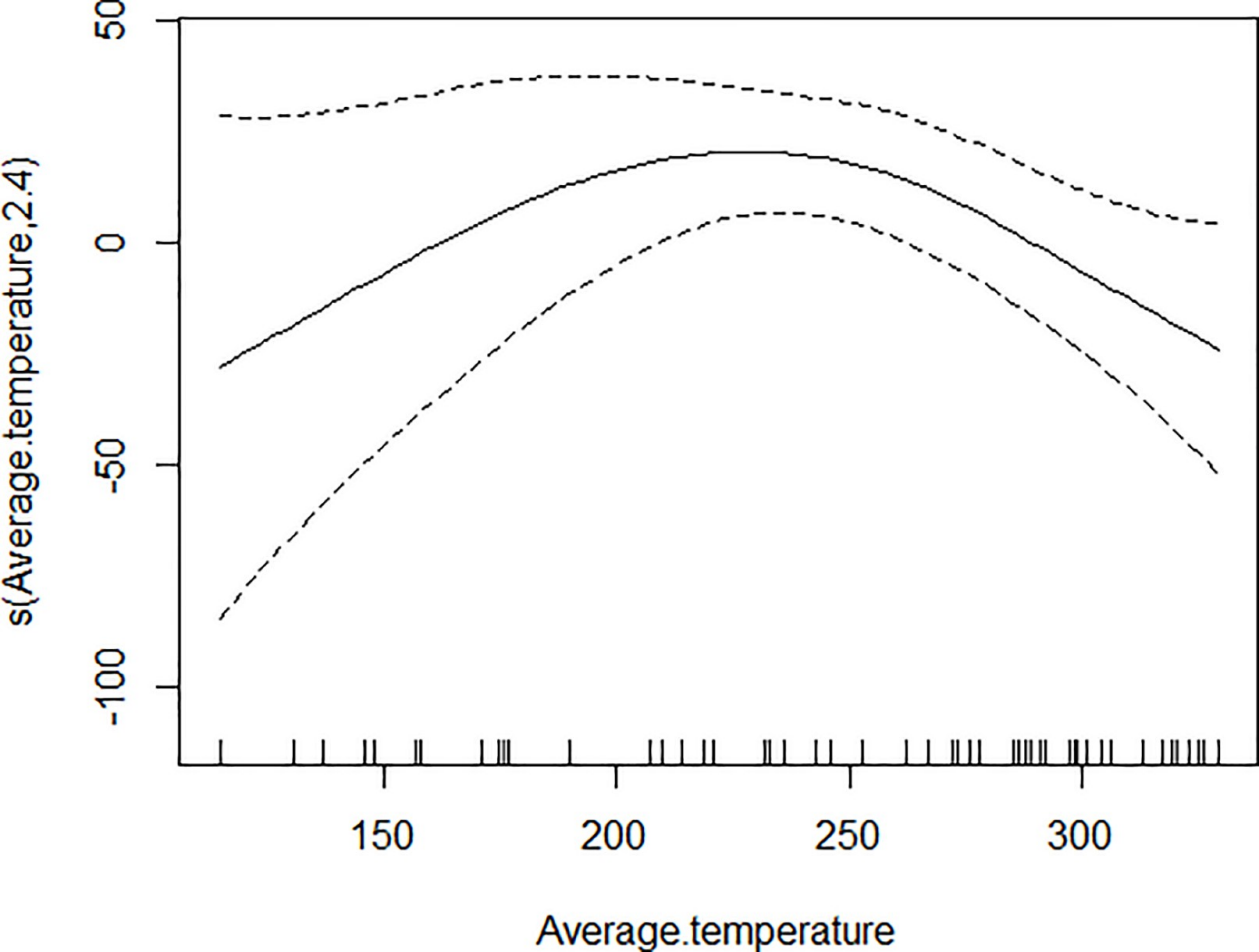
The relationship between BI and average temperature.

**Fig 2:**
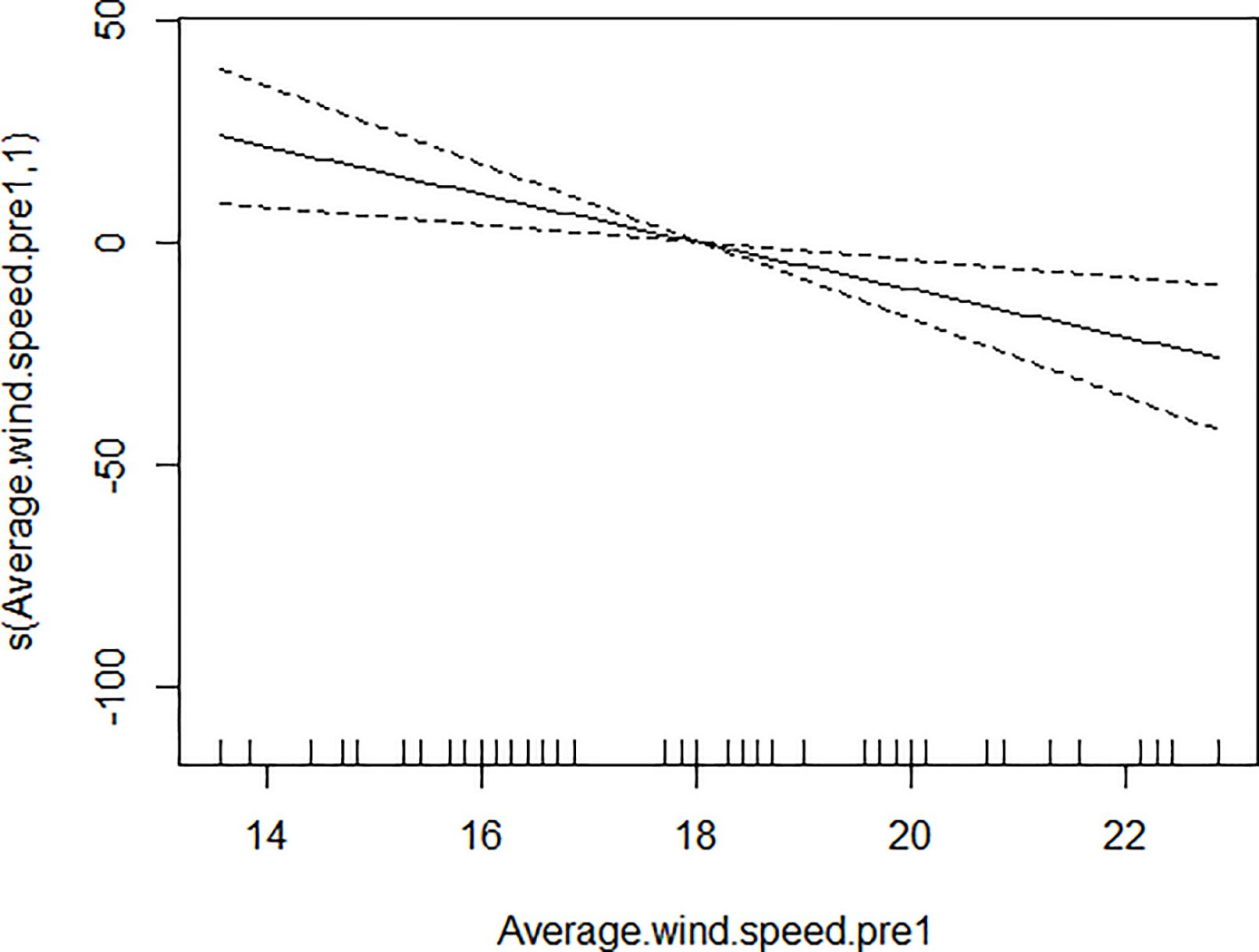
The relationship between BI and average wind speed pre 1 week.

**Fig 3:**
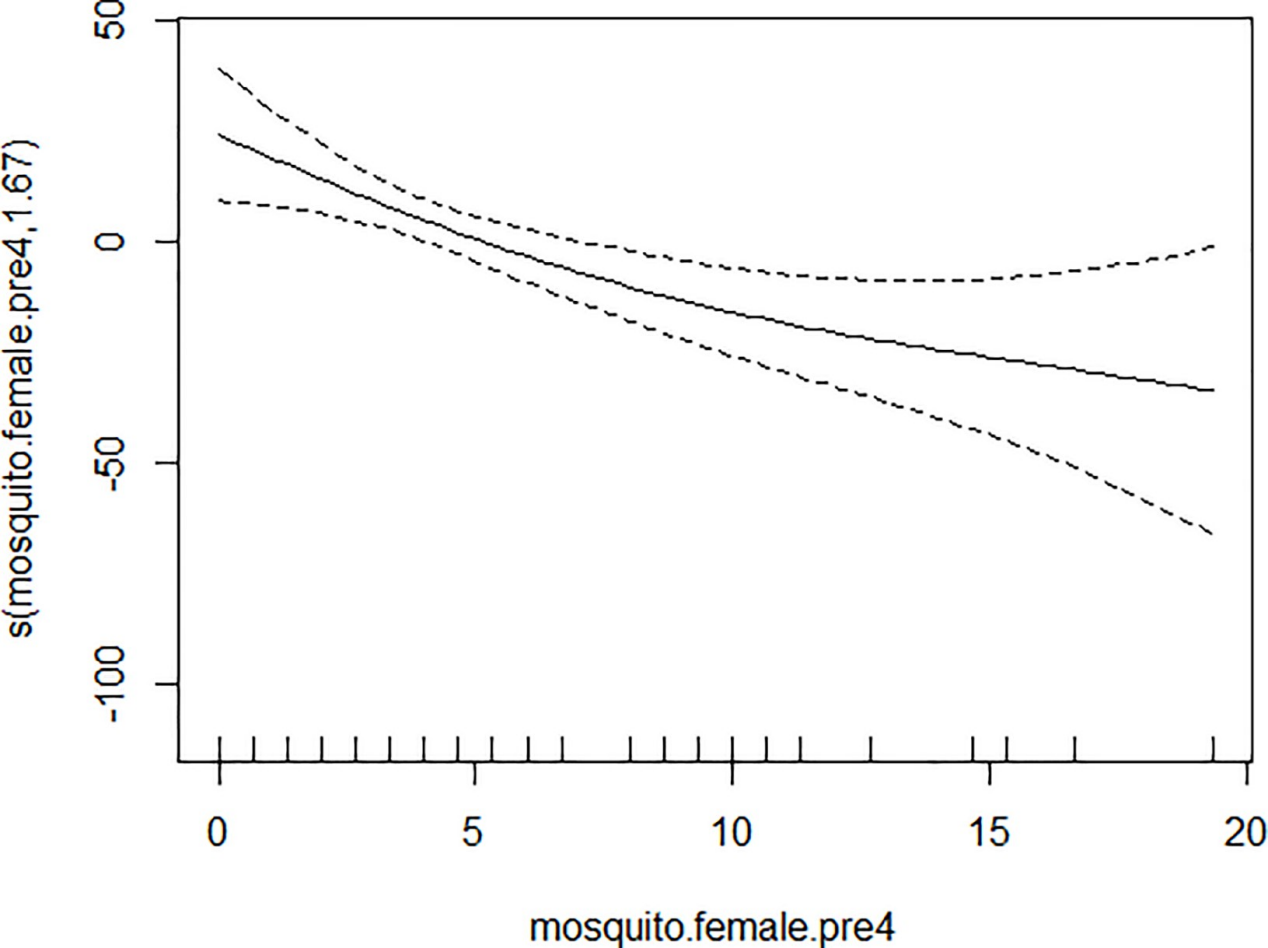
The relationship between BI and female *Ae*. *albopictus* density pre 4 weeks.

**Fig 4:**
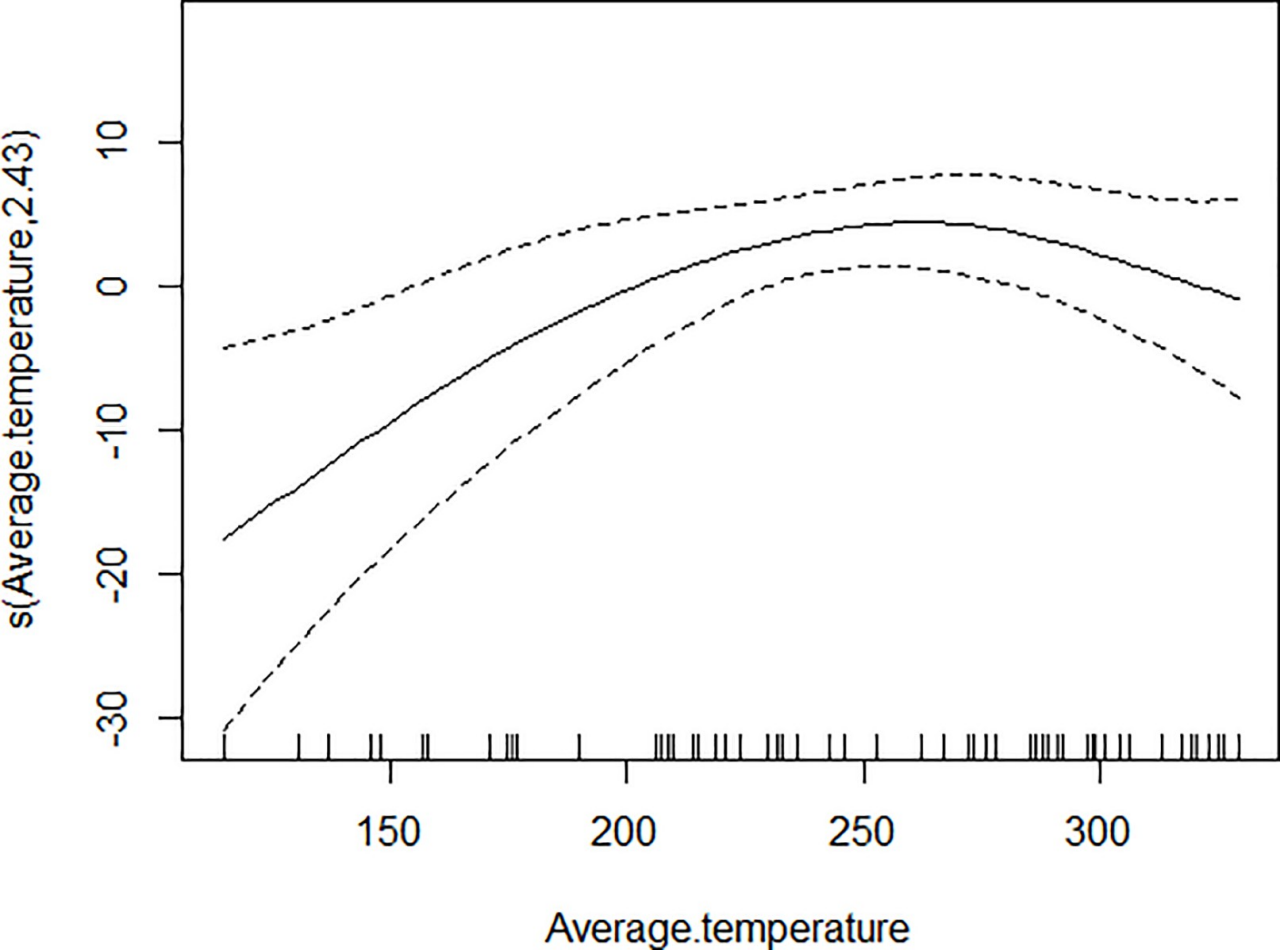
The relationship between CI and average temperature.

**Fig 5:**
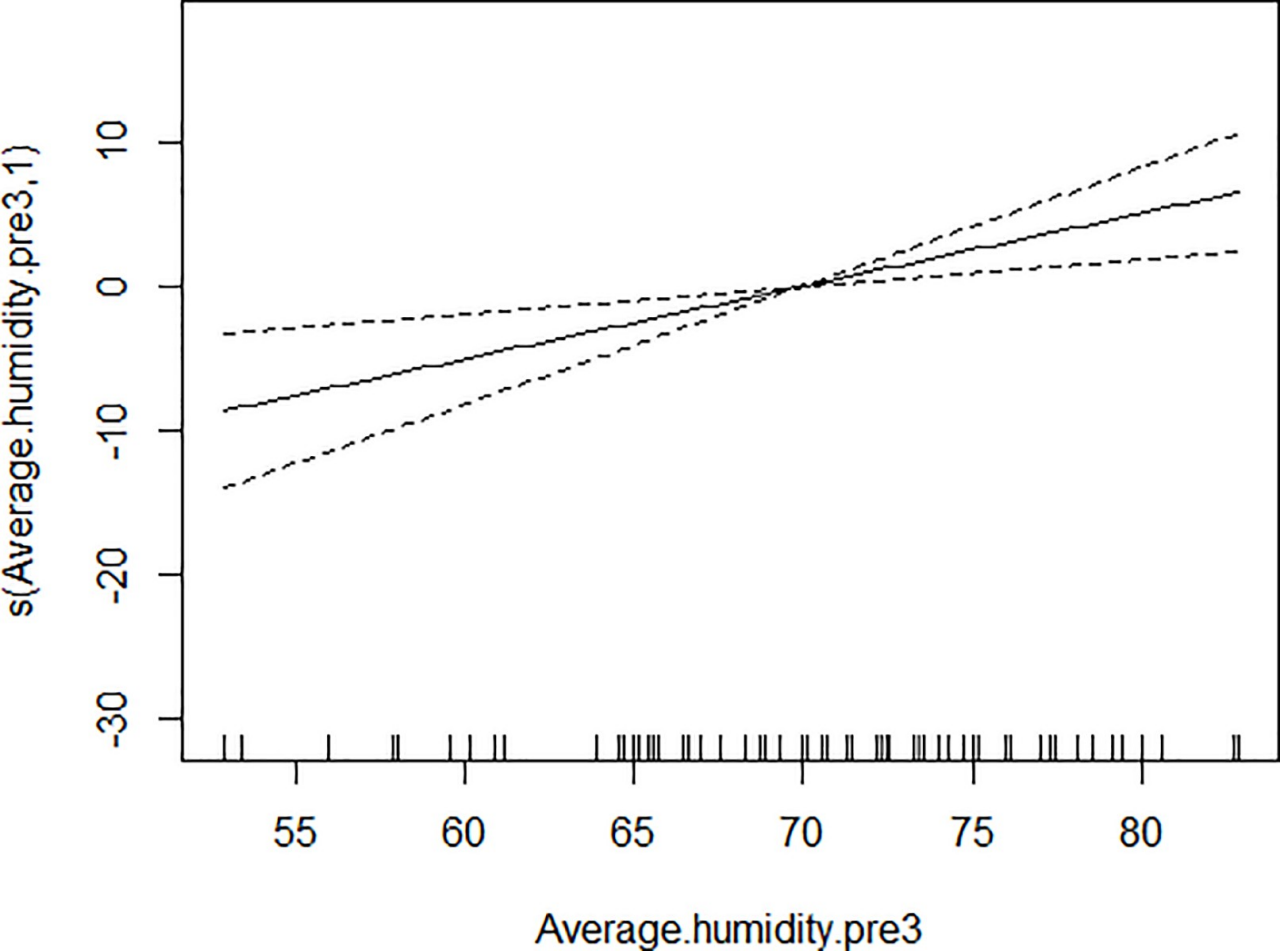
The relationship between CI and average humidity pre 3 weeks.

**Fig 6:**
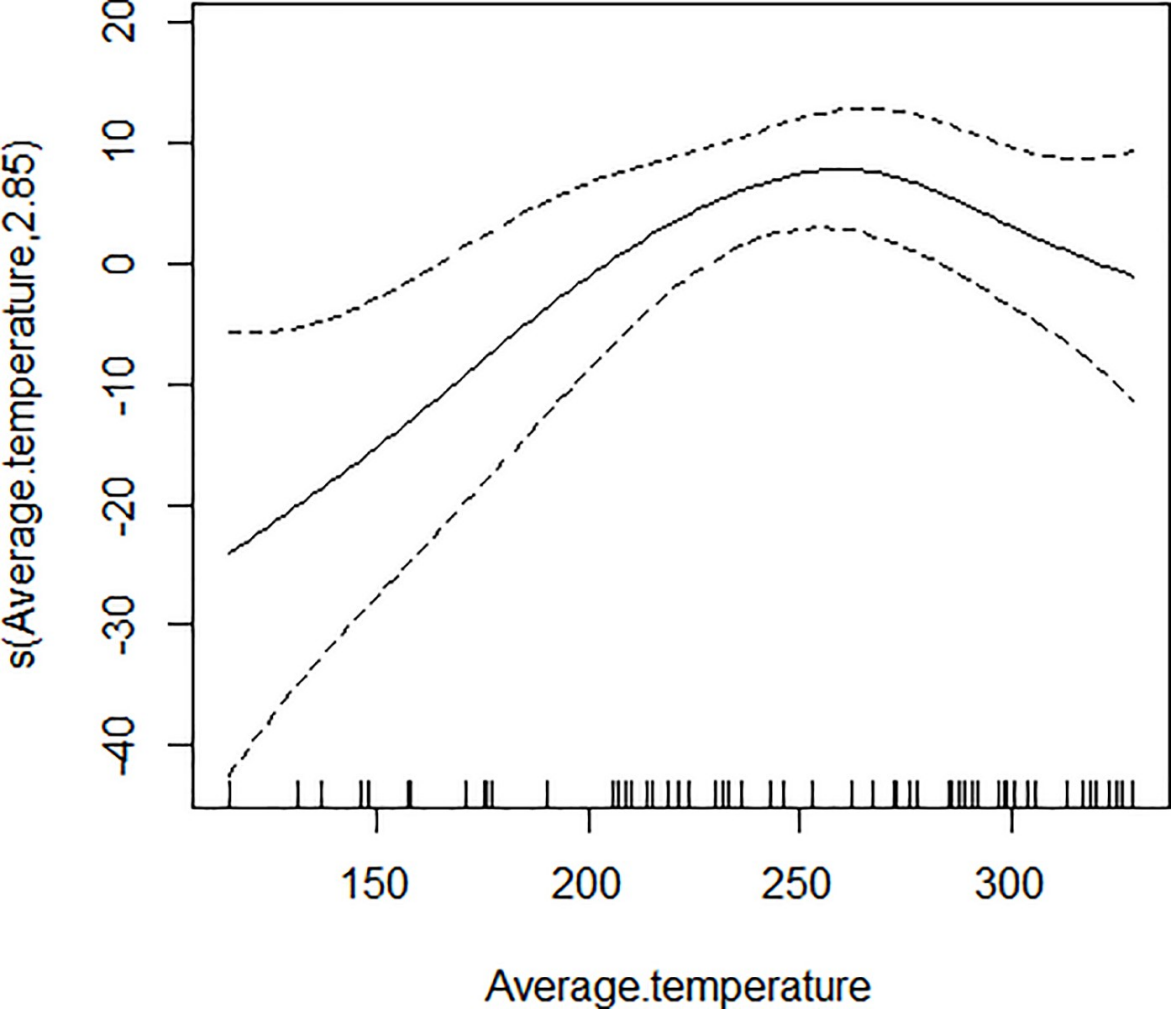
The relationship between HI and average temperature.

**Fig 7:**
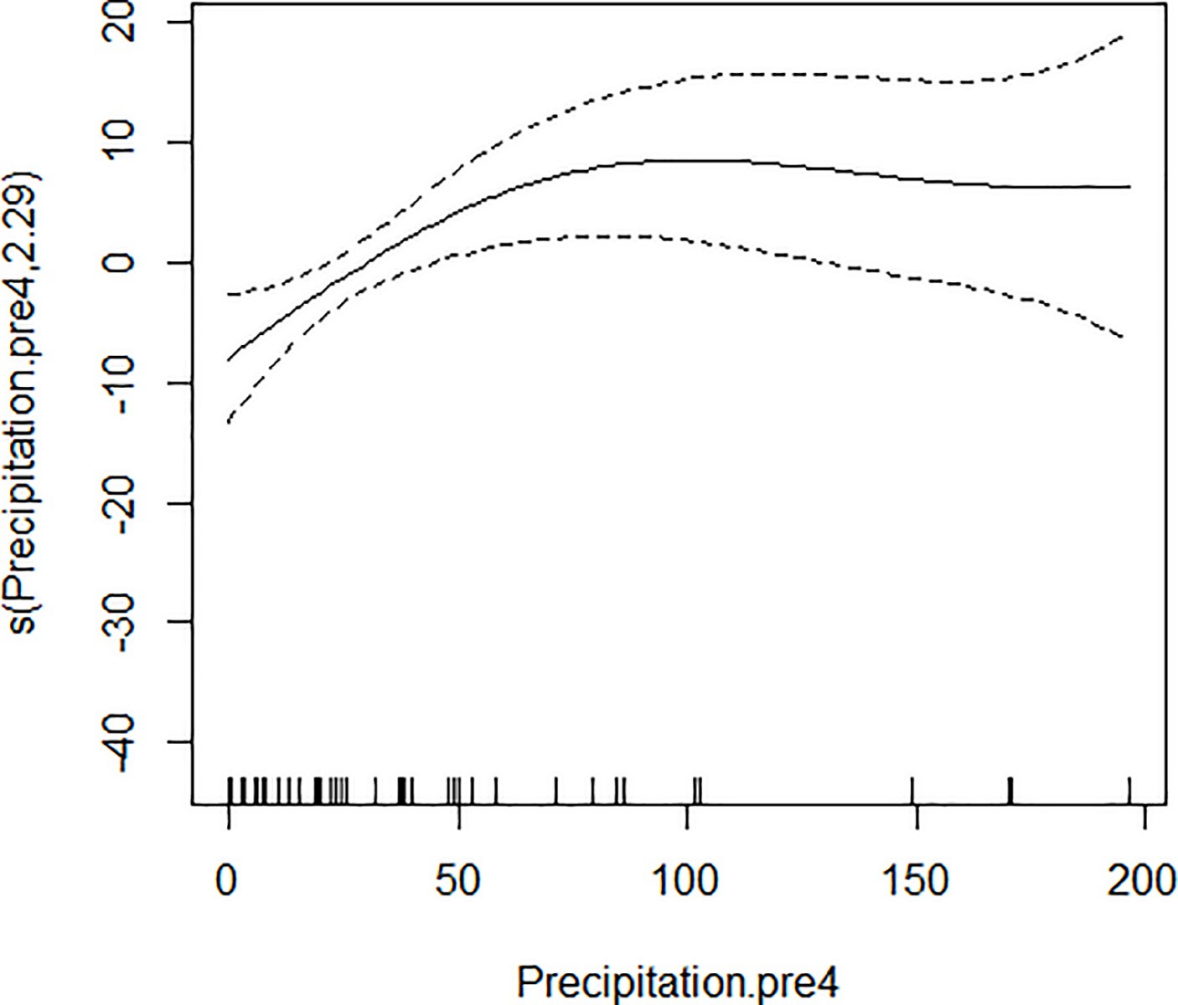
The relationship between HI and precipitation pre 4 weeks.

**Fig 8:**
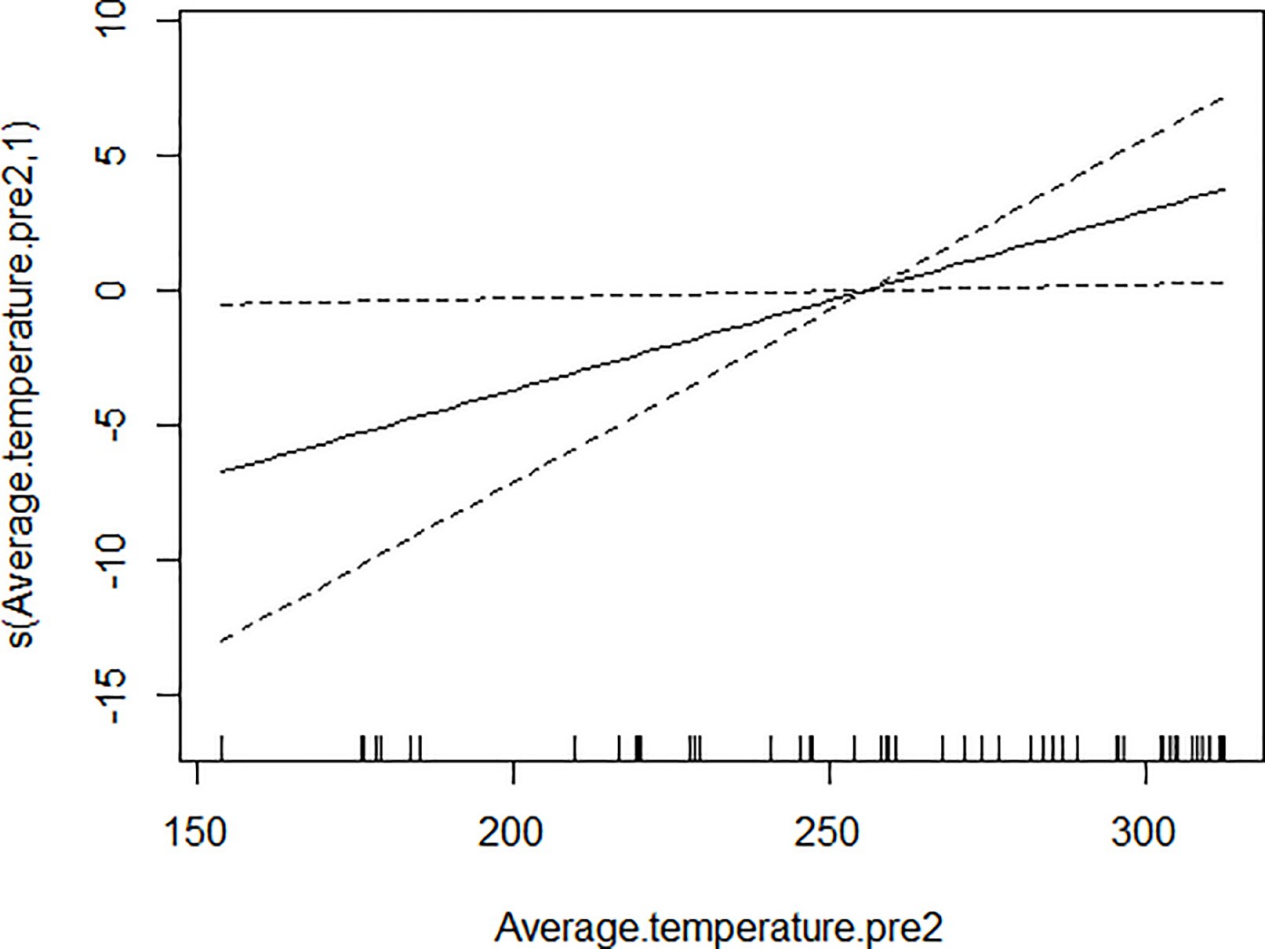
The relationship between *Ae*. *albopictus* density and average temperature pre 2 weeks.

**Fig 9:**
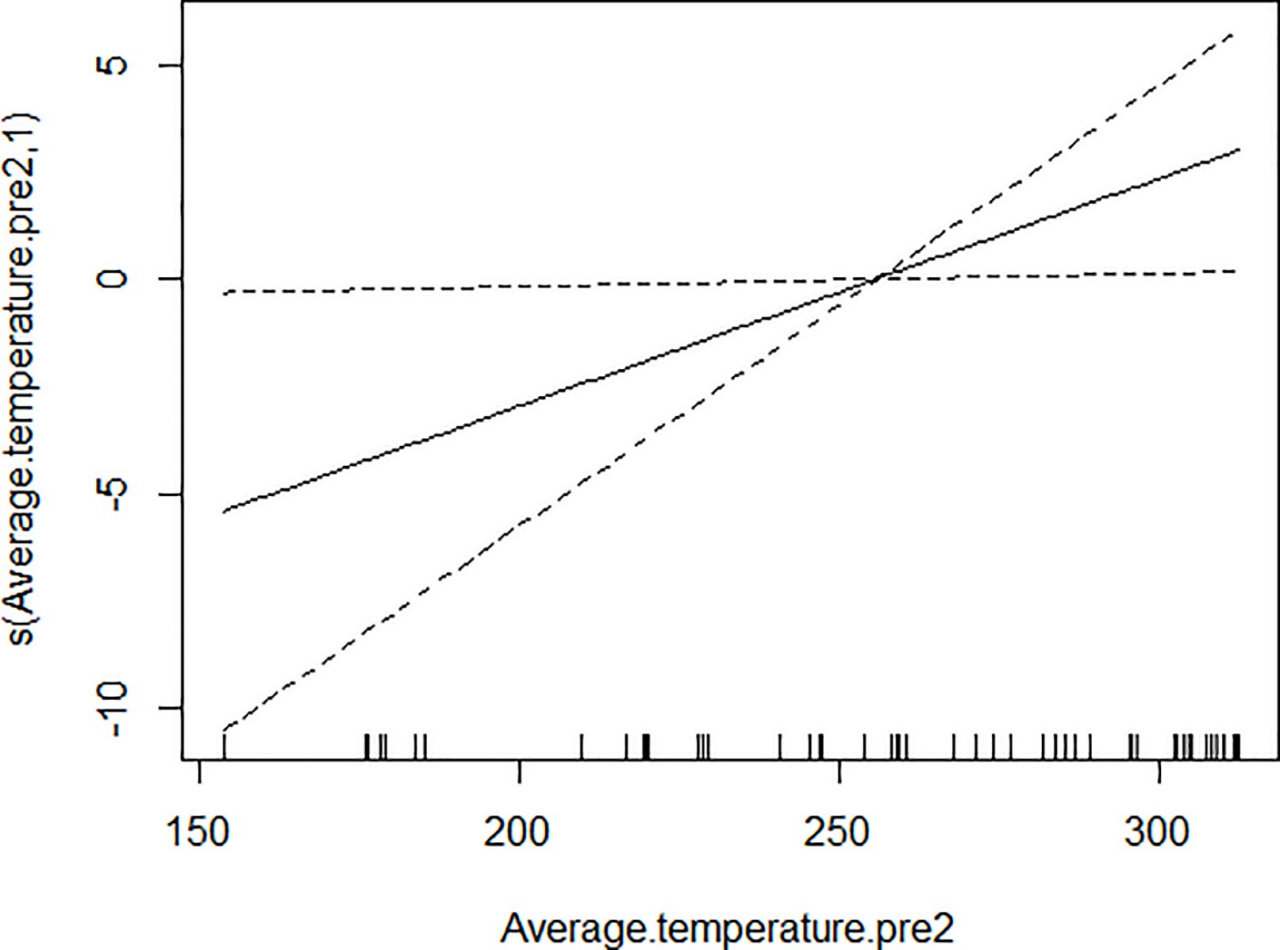
The relationship between female *Ae*. *albopictus* density and average temperature pre 2 weeks.

GAM models were used to analyze the influencing factors related to different density indices of *Ae*. *albopictus*. The significant variables in the correlation analysis were included in the models, and the best effect time of the same variable was selected with the highest correlation coefficient. Although there were high correlation among BI, CI and HI, they were different aspects of the larval density, and consequently the three indices were not included in the model of each other. As shown in Table 3, BI was significantly associated with average temperature, average wind speed pre 1 week and female *Ae*. *albopictus* density pre 4 weeks. BI increased to a peak value first, and then decreased with the increasing of the average temperature (Fig 1), decreased with the increasing of the average wind speed pre 1 week straightly (Fig 2), and decreased smoothly with the increasing of the female *Ae*. *albopictus* density pre 4 weeks (Fig 3). CI was significantly associated with average temperature and average humidity pre 3 weeks. CI increased to a peak value first, and then decreased with the increasing of the average temperature (Fig 4), and increased straightly with the increasing of the average humidity pre 3 weeks (Fig 5). HI was significantly associated with average temperature and precipitation pre 4 weeks. The relationship between HI and the temperature was similar to those with BI and CI (Fig 6), and with the increase of precipitation 4 week ago, HI increased first, then reached a plateau period (Fig 7). The *Ae*. *albopictus* density or female *Ae*. *albopictus* density had linear relationship with the average temperature with a time lag of two weeks (Figs 8 and 9).

**Table 3:** The results of GAM.

Outcomes	Variables	Edf	Linear	*β*	$S_{\bar{x}}$	F/t	*P*
BI	Intercept	—	—	174.789	31.467	5.555	<0.001
	Average air pressure	2.994	No	—	—	2.258*	0.082
	Average wind speed pre1 week	1.000	Yes	-5.341	1.692	-3.157	0.003
	Average temperature	2.405	No	—	—	3.707*	0.018
	Precipitation pre 4 weeks	1.000	Yes	0.070	0.090	0.780	0.440
	Female *Ae*. *albopictus* pre 4 weeks	1.670	No	—	—	6.767*	0.003
CI	Intercept	—	—	567.478	295.566	1.920	0.060
	Average air pressure	1.000	Yes	-0.057	0.029	-1.944	0.057
	Precipitation pre 3 weeks	1.000	Yes	0.031	0.025	1.242	0.220
	Average humidity pre 3 weeks	1.000	Yes	0.507	0.158	3.210	0.002
	Average temperature	2.433	No	—	—	4.449*	0.007
	*Ae*. *albopictus*	2.582	No	—	—	1.188*	0.331
HI	Intercept	—	—	-91.525	470.295	-0.195	0.846
	Average air pressure	1.000	Yes	0.013	0.047	0.286	0.776
	Average temperature	2.848	No	—	—	4.504*	0.005
	Precipitation pre 4 weeks	2.292	No	—	—	3.736*	0.020
*Ae*. *albopictus*	Intercept	—	—	-11.696	10.060	-1.163	0.252
	Average temperature pre 2 weeks	1.000	Yes	0.066	0.031	2.144	0.038
	Average wind speed pre 1 week	1.000	Yes	0.127	0.341	0.372	0.712
	Average air pressure pre 4 weeks	4.022	No	—	—	2.214*	0.072
	Sunshine hours pre 1 week	5.504	No	—	—	1.238*	0.312
	CI pre3	1.000	Yes	-0.024	0.069	-0.344	0.733
Female *Ae*. *albopictus*	Intercept	—	—	-6.075	6.596	-0.921	0.362
	Average temperature pre 2 weeks	1.000	Yes	0.054	0.025	2.154	0.036
	Average wind speed pre 2 weeks	2.153	No	—	—	0.906*	0.573
	Average air pressure pre 4 weeks	2.887	No	—	—	1.036*	0.496
	Sunshine hours pre 1 week	1.000	Yes	-0.006	0.024	-0.252	0.802
	CI pre 3 weeks	1.000	Yes	-0.058	0.054	-1.071	0.290

Edf: degree of freedom. Linear: a linear relationship.*β*: regression coefficient. $S_{\bar{x}}$: standard error of mean.

F/t: the results of ANOVA / T test. *P*: Probability.

*Stands for the ANOVA results.

## Discussion

In the field survey, we found that the *Ae*. *albopictus* density had low correlation with CI or with a time lag of one or three weeks. BI had correlation with female *Ae*. *albopictus* density with a time lag of 4 weeks. The average temperature, precipitation, average humidity, and average wind speed played significant roles in the density of adult mosquito or larva with or without a time lag.

BI is considered as a decision making parameter for mosquito control and dengue epidemic risk. Generally, the BI value of 5 serves as the lowest threshold. In a scenario where the BI value > 5 with reported dengue cases or BI > 20 even without any dengue case, control measures should be taken [18]. Three different risks of HI, with <0.1% as low, 0.1–5% as medium and >5% as high, were suggested by the Pan American Health Organization to prevent dengue transmission [23]. As for CI, one study found that 11.7 was the optimal cut-off value for discriminating outbreaks of dengue [24]. In this study, we found the average BI value was extremely high (86.25) in two villages, and similar values were also seen in HI (42.79%) and CI (30.46%). Although reasons for the high estimates in our study were complicated, there was possible explanation with respect to the breeding place for *Ae*. *albopictus*. As the *Ae*. *albopictus* generally breed in artificial water containers, any type of water-holding container with clean water would be a good larval habitat [3, 8]. The two villages investigated in this study had good sanitation conditions, and vegetation was abundant in and around the villages. Besides, considerable idle containers and water cisterns with clean water were put in or around the yard (accounting for 95.4% of the total number of water containers), which would provide perfect breeding place for *Ae*. *albopictus*. Furthermore, consistent with a previous study [16], the positive rate for *Aedes* larval was found to be higher in discarded tires.

As for adult *Ae*. *albopictus* monitoring, an effective trap would be less intrusive, labor saving, and more comprehensive coverage with an effective lure or attractant. The BG-trap, using CO_2_ and the BG-lure to capture host-seeking female mosquitoes, is an effective mosquito monitoring method. Our entomological survey was conducted at the peak period of *Ae*. *albopictus* density [25], which were representative to a certain extent. The results showed that 86.18% of the adult mosquitoes captured by BG-traps were *Ae*. *albopictus*, indicating that the BG-traps were sensitive for *Ae*. *albopictus*. Consistent with a previous study [9], the BG-traps were more effective in capturing female rather than male *Ae*. *Albopictus* (82.42% vs. 17.58%).

The thresholds of the classical larval indices for management of dengue epidemics were considered to be less effective and sometimes remained poor in predicting adult emergence [18]. Measuring adult mosquito density was the most representative quantitative estimate to obtain data about mosquito abundance, as larva needed to go through several developmental stages to become adult mosquitoes before they could transmit dengue virus [26]. Study had found that the household larval surveys and trap based surveillance systems were not interchangeable approaches [27]. In our study, the *Ae*. *albopictus* density and female *Ae*. *albopictus* density were calculated as two indices and the results were not exactly the same. The *Ae*. *albopictus* density, contained all the captured *Ae*. *albopictus* including male and female, while the female *Ae*. *albopictus* density, calculated the female *Ae*. *albopictus* only. The correlation analysis indicated that the two indices all were slightly correlated with CI with a certain time lag. While the female *Ae*. *albopictus* density pre 4 weeks was negatively correlated with BI, which was consistent with the results of the GAM model but contrary to our common sense. As only female *Ae*. *albopictus* was responsible for disease transmission, the indices would be more appropriate towards female mosquitoes directly. One interesting phenomenon found in our study was that, when the BG-traps were put in the grass or small bamboo grove, more mosquitoes would be caught and most of them were male. These findings may lead to bias of the result for different sites the traps placed, and the different emergence time of the male and female mosquito [9]. Consequently, regarding the correlation between the larval and adult mosquito density, it would be more appropriate towards *Ae*. *albopictus* density than female *Ae*. *albopictus* density.

Climatic factors, particularly the temperature, precipitation and humidity, could directly and indirectly affect the mosquito density and blood feeding behavior [8, 28–29]. In our study, the average temperature was the main influencing factor of the mosquito density, affecting all the study indices. Temperature is crucial for mosquitoes, not only for survival rate but also the lifecycle of the vector including oviposition, hatching, pupation, and emergence processes [16, 30–31]. Higher temperature could reduce the development time of mosquitoes, and increase the propagation speed of the virus [32–34]. Consistent with the above study, our results showed that the adult mosquito density increased straightly with the increase of the average temperature pre two weeks. Studies also found that the effects of temperature on the mortality rate of larvae, pupae and adult mosquitoes could be U-shaped with a lower mortality rate was seen when temperature ranged from 15 to 30°C [20–21, 35]. This probably explained the decrease of the BI, CI and HI from the peak value along with the increase of the average temperature in our study.

Precipitation played a crucial role in the transmission of mosquito borne diseases, due to the fact that mosquito required water for the aquatic larval and pupal breeding stages. Higher pupal productivity and entomological indices was found in the rainy season than dry season [3, 16, 26], and the effect of the precipitation to the larval density may have a time lag from 2 months to 1 month [33]. Precipitation could also influence the adult mosquito capturing. One study found that the BGS traps consistently captured nearly 20% of the marked female *Aedes* population in the wet season and about 30% in the hot and dry season [36]. Besides, BGS traps could increase the biting rate of mosquito via increasing the contact between humans and mosquitoes, as humans often stayed indoors when it rained [4]. Based on our results, with the increase of precipitation 4 weeks ago, HI increased at the beginning, and then reached a plateau period. Less precipitation reduced amount of water retained in containers which affect mosquito breeding. However, extremely heavy precipitation could lead to water containers saturation or even flush mosquito larvae away from breeding sites, eliminating habitats to decrease the vector population [26], which possible explained the plateaus of HI.

Relative humidity was an important meteorological factor in the life-cycle of mosquitoes [15], especially in lowland plains [16]. Humidity could also increase the transmission rate of human dengue fever infection in the context of imported dengue cases and mosquito density [4, 30]. Relative humidity could affect larvae density by affecting adult mosquito survival, and also had a synergistic effect with the temperature [17]. While in our GAM models, CI increased with the rise of the average humidity pre 3 weeks. The wind speed could influence the effectiveness of the daily captures of mosquitoes [37]. Yin Q et al. suggested that the predicted hourly *Ae*. *albopictus* densities generally decreased with wind speed [25]. Endo N et al. found that wind direction and speed could influence the malaria vector populations by affecting the effect of CO_2_ attraction and enable mosquitoes to identify village location [38]. In our models, the average wind speed was negatively correlated with BI and with one week lag effect. Higher wind speed may affect the dynamics of the mosquito population by affecting wave activity, advection of adult mosquitoes, and CO_2_ attraction, resulting in a low density of larvae after a period of time.

Our study had several strengths. This is one of the few studies investigating the relationships between larval indices and the adult mosquito with BG-trap method in mainland China. Besides, during the analysis procedure, various meteorological factors were taken into consideration in our study. Meanwhile, some limitations must be recognized in this study. Firstly, as the study sites and samples were only selected from rural area of Zhejiang Province, our results cannot be generalisable to broader national level. Secondly, the study relationships may be confounded by other factors such as socio-economic characteristics and human activity, which were not included in the current analysis.

## Conclusions

Our findings suggested that the BG-trap was an effective adult trap for *Ae*. *albopictus*, especially for the female mosquitoes. The adult *Ae*. *albopictus* density was slightly correlated with certain larval indices. The average temperature, precipitation, average humidity, and average wind speed played significant roles in the density of adult mosquito or larva with or without a time lag. To prevent dengue fever, new monitoring method and thresholds should be developed based on adult mosquitoes, with considering meteorological factors.

## Supporting information

S1 Data (XLS)
